# Supplementary material for: New footprints from Laetoli (Tanzania) provide evidence for marked body size variation in early hominins
Source: eLife. 2016 Dec 14;5:e19568. doi: 10.7554/eLife.19568 (PMC5156529; doi:10.7554/eLife.19568)
Supplement: Supplementary file 1. — DOI: http://dx.doi.org/10.7554/eLife.19568.022 [file elife-19568-supp1.docx]

**Supplementary file 1.** Footprint imaging, measurement report 1.

Fieldwork measurement acquisition and error calculation.

| **ID TRENCH** | **ID MEASURE** | **ID 1° TARGET** | **H 1° TARGET (m)** | **ID 2° TARGET** | **H 2° TARGET (m)** | **DISTANCE (m)** | **Δ MEASURED (m)** | **Δ CORRECTED (m)** |  |
| --- | --- | --- | --- | --- | --- | --- | --- | --- | --- |
| L8 | 1 | A | 0.775 | B | 0.725 | 2.561 | 0.050 | 0.051 |  |
| L8 | 2 | B | 0.774 | C | 0.921 | 3.271 | -0.147 | -0.146 |  |
| L8 | 3 | C | 0.486 | D | 0.613 | 3.441 | -0.127 | -0.126 |  |
| L8 | 4 | D | 0.702 | A | 0.482 | 3.591 | 0.220 | 0.221 |  |
| L8 | 5 | A | 0.523 | C | 0.620 | 4.176 | **ERROR (m)** | **ERROR DISTRIBUT. (m)** | **FINAL ERROR (m)** |
| L8 | 6 | B | 0.453 | D | 0.724 | 4.894 | -0.004 | -0.001 | 0.000 |
|  |  |  |  |  |  |  |  |  |  |
| M9 | 7 | E | 0.660 | F | 0.622 | 2.335 | 0.038 | 0.038 |  |
| M9 | 8 | F | 0.705 | G | 0.690 | 2.861 | 0.015 | 0.015 |  |
| M9 | 9 | G | 0.736 | H | 0.720 | 2.884 | 0.016 | 0.016 |  |
| M9 | 10 | H | 0.799 | E | 0.867 | 3.951 | -0.068 | -0.068 |  |
| M9 | 11 | E | 0.745 | G | 0.690 | 4.276 | **ERROR (m)** | **ERROR DISTRIBUT. (m)** | **FINAL ERROR (m)** |
| M9 | 12 | F | 0.808 | H | 0.765 | 4.209 | 0.001 | 0.000 | 0.000 |
|  |  |  |  |  |  |  |  |  |  |
| TP2 | 13 | I | 0.581 | J | 0.600 | 1.333 | -0.019 | -0.020 |  |
| TP2 | 14 | J | 0.587 | K | 0.548 | 1.581 | 0.039 | 0.039 |  |
| TP2 | 15 | K | 0.549 | L | 0.518 | 1.444 | 0.031 | 0.031 |  |
| TP2 | 16 | L | 0.477 | I | 0.526 | 1.831 | -0.049 | -0.050 |  |
| TP2 | 17 | I | 0.517 | K | 0.498 | 2.231 | **ERROR (m)** | **ERROR DISTRIBUT. (m)** | **FINAL ERROR (m)** |
| TP2 | 18 | J | 0.544 | L | 0.469 | 2.169 | 0.002 | 0.000 | 0.000 |
|  |  |  |  |  |  |  |  |  |  |
| M10 | 19 | M | 0.701 | N | 0.686 | 2.211 | 0.015 | 0.015 |  |
| M10 | 20 | N | 0.658 | O | 0.578 | 3.696 | 0.080 | 0.081 |  |
| M10 | 21 | O | 0.609 | P | 0.614 | 2.304 | -0.005 | -0.004 |  |
| M10 | 22 | P | 0.566 | M | 0.658 | 3.621 | -0.092 | -0.092 |  |
| M10 | 23 | M | 0.659 | O | 0.562 | 4.291 | **ERROR (m)** | **ERROR DISTRIBUT. (m)** | **FINAL ERROR (m)** |
| M10 | 24 | N | 0.645 | P | 0.564 | 4.306 | -0.002 | -0.001 | 0.000 |
