# Supplementary material for: New footprints from Laetoli (Tanzania) provide evidence for marked body size variation in early hominins
Source: eLife. 2016 Dec 14;5:e19568. doi: 10.7554/eLife.19568 (PMC5156529; doi:10.7554/eLife.19568)
Supplement: Supplementary file 2. — DOI: http://dx.doi.org/10.7554/eLife.19568.023 [file elife-19568-supp2.docx]

**Supplementary file 2.** Footprint imaging, measurement report 2.

STAR*NET reports of measurements in plan and altitude and calculation of the new adjusted x,y,z-coordinates.

| **STAR*NET REPORT OF L8 (PLAN)** | | | | | | |
| --- | --- | --- | --- | --- | --- | --- |
| **Adjustment Statistical Summary** | | | | | | |
| Iterations | | 2 |  |  |  |  |
| Number of Stations | | 4 |  |  |  |  |
| Number of Observations | | 11 |  |  |  |  |
| Number of Unknowns | | 5 |  |  |  |  |
| Number of Redundant Obs | | 6 |  |  |  |  |
| **Adjusted Station Information** | | | | | | |
| **Adjusted Coordinates (Meters)** | | | | | | |
| **Station** | **E** | **N** |  |  |  |  |
| A | 0.8470 | 0.0000 |  |  |  |  |
| B | 3.4125 | 0.0000 |  |  |  |  |
| C | 3.4331 | 3.2739 |  |  |  |  |
| D | -0.0041 | 3.4941 |  |  |  |  |
| **Adjusted Observations and Residuals** | | | | | | |
| **Adjusted Coordinate Observations (Meters) (Stations with Partially Fixed Coordinate Components)** | | | | | | |
| **Station** | **Component** | **Adj Coordinate** | **Residual** | **StdErr** | **StdRes** | **File:Line** |
| C | E | 3.4331 | -0.0099 | 0.0300 | 0.3 | 1:3 |
|  | N | 3.2739 | 0.0029 | 0.0300 | 0.1 |  |
| D | E | -0.0041 | -0.0041 | 0.0300 | 0.1 | 1:4 |
|  | N | 3.4941 | -0.0029 | 0.0300 | 0.1 |  |
| B | E | 3.4125 | 0.0045 | 0.0300 | 0.2 | 1:2 |
| **Adjusted Distance Observations (Meters)** | | | | | | |
| **From** | **To** | **Distance** | **Residual** | **StdErr** | **StdRes** | **File:Line** |
| B | D | 4.8869 | -0.0071 | 0.0091 | 0.8 | 1:16 |
| D | A | 3.5963 | 0.0053 | 0.0091 | 0.6 | 1:13 |
| A | B | 2.5655 | 0.0045 | 0.0091 | 0.5 | 1:7 |
| A | C | 4.1721 | -0.0039 | 0.0091 | 0.4 | 1:15 |
| C | D | 3.4443 | 0.0033 | 0.0091 | 0.4 | 1:11 |
| B | C | 3.274 | 0.003 | 0.0091 | 0.3 | 1:9 |
|  |  |  |  |  |  |  |
| **STAR*NET REPORT OF L8 (ALTITUDE)** | | | | | | |
| **Adjustment Statistical Summary** | | | | | | |
| Number of Stations | | 4 |  |  |  |  |
| Number of Observations | | 6 |  |  |  |  |
| Number of Unknowns | | 3 |  |  |  |  |
| Number of Redundant Obs | | 3 |  |  |  |  |
| **Adjusted Station Information** | | | | | | |
| **Adjusted Elevations and Error Propagation (Meters)** | | | | | | |
| **Station** | **Elev** | **StdDev** | **95** |  |  |  |
| A | 1.0000 | 0.0000 | 0.0000 |  |  |  |
| B | 1.0503 | 0.0010 | 0.0019 |  |  |  |
| C | 0.9040 | 0.0010 | 0.0020 |  |  |  |
| D | 0.7787 | 0.0010 | 0.0021 |  |  |  |
| **Adjusted Observations and Residuals** | | | | | | |
| **Adjusted Differential Level Observations (Meters)** | | | | | | |
| **From** | **To** | **Elev Diff** | **Residual** | **StdErr** | **StdRes** | **File:Line** |
| C | D | -0.1253 | 0.0017 | 0.0003 | 5.6* | 1:8 |
| D | A | 0.2213 | 0.0013 | 0.0003 | 4.3* | 1:9 |
| A | C | -0.0960 | 0.0010 | 0.0003 | 3.2* | 1:10 |
| B | C | -0.1462 | 0.0008 | 0.0003 | 2.6 | 1:7 |
| B | D | -0.2716 | -0.2716 | 0.0003 | 1.7 | 1:11 |
| A | B | 0.0503 | 0.0503 | 0.0003 | 1.1 | 1:6 |

| **STAR*NET REPORT OF M9 (PLAN)** | | | | | | |
| --- | --- | --- | --- | --- | --- | --- |
| **Adjustment Statistical Summary** | | | | | | |
| Iterations | | 2 |  |  |  |  |
| Number of Stations | | 4 |  |  |  |  |
| Number of Observations | | 11 |  |  |  |  |
| Number of Unknowns | | 5 |  |  |  |  |
| Number of Redundant Obs | | 6 |  |  |  |  |
| **Adjusted Station Information** | | | | | | |
| **Adjusted Coordinates (Meters)** | | | | | | |
| **Station** | **E** | **N** |  |  |  |  |
| E | 0.0000 | 0.0000 |  |  |  |  |
| F | 2.3344 | 0.0000 |  |  |  |  |
| G | 3.3322 | 2.6807 |  |  |  |  |
| H | 0.7124 | 3.8853 |  |  |  |  |
| **Adjusted Observations and Residuals** | | | | | | |
| **Adjusted Coordinate Observations (Meters) (Stations with Partially Fixed Coordinate Components)** | | | | | | |
| **Station** | **Component** | **Adj Coordinate** | **Residual** | **StdErr** | **StdRes** | **File:Line** |
| G | E | 3.3322 | 0.0022 | 0.0300 | 0.1 | 1:3 |
|  | N | 2.6807 | -0.0013 | 0.0300 | 0.0 |  |
| H | E | 0.7124 | 0.0014 | 0.0300 | 0.0 | 1:4 |
|  | N | 3.8853 | 0.0003 | 0.0300 | 0.0 |  |
| F | E | 2.3344 | -0.0006 | 0.0300 | 0.0 | 1:2 |
| **Adjusted Distance Observations (Meters)** | | | | | | |
| **From** | **To** | **Distance** | **Residual** | **StdErr** | **StdRes** | **File:Line** |
| F | H | 4.2102 | 0.0012 | 0.0091 | 0.1 | 1:11 |
| H | E | 3.9500 | -0.0010 | 0.0091 | 0.1 | 1:9 |
| E | G | 4.2767 | 0.0007 | 0.0091 | 0.1 | 1:10 |
| E | F | 2.3344 | -0.0006 | 0.0091 | 0.1 | 1:6 |
| F | G | 2.8604 | -0.0006 | 0.0091 | 0.1 | 1:7 |
| G | H | 2.8834 | -0.0006 | 0.0091 | 0.1 | 1:8 |
|  |  |  |  |  |  |  |
| **STAR*NET REPORT OF M9 (ALTITUDE)** | | | | | | |
| **Adjustment Statistical Summary** | | | | | | |
| Number of Stations | | 4 |  |  |  |  |
| Number of Observations | | 6 |  |  |  |  |
| Number of Unknowns | | 3 |  |  |  |  |
| Number of Redundant Obs | | 3 |  |  |  |  |
| **Adjusted Station Information** | | | | | | |
| **Adjusted Elevations and Error Propagation (Meters)** | | | | | | |
| **Station** | **Elev** | **StdDev** | **95** |  |  |  |
| E | 1.000000 | 0.000002 | 0.000003 |  |  |  |
| F | 1.036200 | 0.003261 | 0.006392 |  |  |  |
| G | 1.053900 | 0.003580 | 0.007018 |  |  |  |
| H | 1.072000 | 0.003644 | 0.007142 |  |  |  |
| **Adjusted Observations and Residuals** | | | | | | |
| **Adjusted Differential Level Observations (Meters)** | | | | | | |
| **From** | **To** | **Elev Diff** | **Residual** | **StdErr** | **StdRes** | **File:Line** |
| F | H | 0.0358 | -0.0072 | 0.0003 | 22.3* | 1:11 |
| H | E | -0.0720 | -0.0040 | 0.0003 | 12.7* | 1:9 |
| F | G | 0.0177 | 0.0027 | 0.0003 | 10.2* | 1:7 |
| G | H | 0.0180 | 0.0020 | 0.0003 | 7.6* | 1:8 |
| E | F | 0.0362 | -0.0018 | 0.0003 | 7.4* | 1:6 |
| E | G | 0.0539 | -0.0011 | 0.0003 | 3.2* | 1:10 |

| **STAR*NET REPORT OF TP2 (PLAN)** | | | | | | |
| --- | --- | --- | --- | --- | --- | --- |
| **Adjustment Statistical Summary** | | | | | | |
| Iterations | | 2 |  |  |  |  |
| Number of Stations | | 4 |  |  |  |  |
| Number of Observations | | 11 |  |  |  |  |
| Number of Unknowns | | 5 |  |  |  |  |
| Number of Redundant Obs | | 6 |  |  |  |  |
| **Adjusted Station Information** | | | | | | |
| **Adjusted Coordinates (Meters)** | | | | | | |
| **Station** | **E** | **N** |  |  |  |  |
| I | 0.0000 | 0.0000 |  |  |  |  |
| J | 1.3348 | 0.0000 |  |  |  |  |
| K | 1.5908 | 1.5616 |  |  |  |  |
| L | 0.1694 | 1.8256 |  |  |  |  |
| **Adjusted Observations and Residuals** | | | | | | |
| **Adjusted Coordinate Observations (Meters) (Stations with Partially Fixed Coordinate Components)** | | | | | | |
| **Station** | **Component** | **Adj Coordinate** | **Residual** | **StdErr** | **StdRes** | **File:Line** |
| K | E | 1.5908 | -0.0052 | 0.0300 | 0.2 | 1:3 |
|  | N | 1.5616 | 0.0026 | 0.0300 | 0.1 |  |
| L | E | 0.1694 | -0.0036 | 0.0300 | 0.1 | 1:4 |
|  | N | 1.8256 | -0.0004 | 0.0300 | 0.0 |  |
| J | E | 1.3348 | 0.0018 | 0.0300 | 0.1 | 1:2 |
| **Adjusted Distance Observations (Meters)** | | | | | | |
| **From** | **To** | **Distance** | **Residual** | **StdErr** | **StdRes** | **File:Line** |
| J | L | 2.1658 | -0.0032 | 0.0091 | 0.3 | 1:11 |
| L | I | 1.8334 | 0.0024 | 0.0091 | 0.3 | 1:9 |
| I | K | 2.2291 | -0.0019 | 0.0091 | 0.2 | 1:10 |
| I | J | 1.3348 | 0.0018 | 0.0091 | 0.2 | 1:6 |
| K | L | 1.4456 | 0.0016 | 0.0091 | 0.2 | 1:8 |
| J | K | 1.5824 | 0.0014 | 0.0091 | 0.2 | 1:7 |
|  |  |  |  |  |  |  |
| **STAR*NET REPORT OF TP2 (ALTITUDE)** | | | | | | |
| **Adjustment Statistical Summary** | | | | | | |
| Number of Stations | | 4 |  |  |  |  |
| Number of Observations | | 6 |  |  |  |  |
| Number of Unknowns | | 3 |  |  |  |  |
| Number of Redundant Obs | | 3 |  |  |  |  |
| **Adjusted Station Information** | | | | | | |
| **Adjusted Elevations and Error Propagation (Meters)** | | | | | | |
| **Station** | **Elev** | **StdDev** | **95** |  |  |  |
| I | 1.000000 | 0.000001 | 0.000002 |  |  |  |
| J | 0.979500 | 0.001645 | 0.003224 |  |  |  |
| K | 1.019200 | 0.001766 | 0.003461 |  |  |  |
| L | 1.051000 | 0.001750 | 0.003431 |  |  |  |
| **Adjusted Observations and Residuals** | | | | | | |
| **Adjusted Differential Level Observations (Meters)** | | | | | | |
| **From** | **To** | **Elev Diff** | **Residual** | **StdErr** | **StdRes** | **File:Line** |
| J | L | 0.0715 | -0.0035 | 0.0002 | 15.0* | 1:11 |
| L | I | -0.0510 | -0.0020 | 0.0002 | 9.2* | 1:9 |
| I | J | -0.0205 | -0.0015 | 0.0002 | 8.4* | 1:6 |
| K | L | 0.0318 | 0.0008 | 0.0002 | 4.1* | 1:8 |
| J | K | 0.0397 | 0.0007 | 0.0002 | 3.6* | 1:7 |
| I | K | 0.0192 | 0.0002 | 0.0002 | 0.8 | 1:10 |

| **STAR*NET REPORT OF M10 (PLAN)** | | | | | | |
| --- | --- | --- | --- | --- | --- | --- |
| **Adjustment Statistical Summary** | | | | | | |
| Iterations | | 1 |  |  |  |  |
| Number of Stations | | 4 |  |  |  |  |
| Number of Observations | | 11 |  |  |  |  |
| Number of Unknowns | | 5 |  |  |  |  |
| Number of Redundant Obs | | 6 |  |  |  |  |
| **Adjusted Station Information** | | | | | | |
| **Adjusted Coordinates (Meters)** | | | | | | |
| **Station** | **E** | **N** |  |  |  |  |
| M | 0.1220 | 0.0000 |  |  |  |  |
| N | 2.3330 | 0.0000 |  |  |  |  |
| O | 2.3025 | 3.6958 |  |  |  |  |
| P | -0.0003 | 3.6190 |  |  |  |  |
| **Adjusted Observations and Residuals** | | | | | | |
| **Adjusted Coordinate Observations (Meters) (Stations with Partially Fixed Coordinate Components)** | | | | | | |
| **Station** | **Component** | **Adj Coordinate** | **Residual** | **StdErr** | **StdRes** | **File:Line** |
| O | E | 2.3025 | -0.0005 | 0.0300 | 0.0 | 1:3 |
|  | N | 3.6958 | -0.0002 | 0.0300 | 0.0 |  |
| P | E | -0.0003 | -0.0003 | 0.0300 | 0.0 | 1:4 |
|  | N | 3.6190 | 0.0000 | 0.0300 | 0.0 |  |
| N | E | 2.3330 | 0.0000 | 0.0300 | 0.0 | 1:2 |
| **Adjusted Distance Observations (Meters)** | | | | | | |
| **From** | **To** | **Distance** | **Residual** | **StdErr** | **StdRes** | **File:Line** |
| M | O | 4.2911 | 0.0001 | 0.0091 | 0.0 | 1:10 |
| N | O | 3.6959 | -0.0001 | 0.0091 | 0.0 | 1:7 |
| N | P | 4.3059 | -0.0001 | 0.0091 | 0.0 | 1:11 |
| P | M | 3.6210 | 0.0000 | 0.0091 | 0.0 | 1:9 |
| M | N | 2.2110 | 0.0000 | 0.0091 | 0.0 | 1:6 |
| O | P | 2.3040 | 0.0000 | 0.0091 | 0.0 | 1:8 |
|  |  |  |  |  |  |  |
| **STAR*NET REPORT OF M10 (ALTITUDE)** | | | | | | |
| **Adjustment Statistical Summary** | | | | | | |
| Number of Stations | | 4 |  |  |  |  |
| Number of Observations | | 6 |  |  |  |  |
| Number of Unknowns | | 3 |  |  |  |  |
| Number of Redundant Obs | | 3 |  |  |  |  |
| **Adjusted Station Information** | | | | | | |
| **Adjusted Elevations and Error Propagation (Meters)** | | | | | | |
| **Station** | **Elev** | **StdDev** | **95** |  |  |  |
| M | 1.000000 | 0.000001 | 0.000001 |  |  |  |
| N | 1.014700 | 0.001340 | 0.002627 |  |  |  |
| O | 1.096700 | 0.001479 | 0.002898 |  |  |  |
| P | 1.092800 | 0.001458 | 0.002857 |  |  |  |
| **Adjusted Observations and Residuals** | | | | | | |
| **Adjusted Differential Level Observations (Meters)** | | | | | | |
| **From** | **To** | **Elev Diff** | **Residual** | **StdErr** | **StdRes** | **File:Line** |
| N | P | 0.0781 | -0.0029 | 0.0003 | 8.9* | 1:11 |
| N | O | 0.0820 | 0.0020 | 0.0003 | 6.6* | 1:7 |
| O | P | -0.0039 | 0.0011 | 0.0003 | 4.5* | 1:8 |
| P | M | -0.0928 | -0.0008 | 0.0003 | 2.5 | 1:9 |
| M | O | 0.0967 | -0.0003 | 0.0003 | 0.9 | 1:10 |
| M | N | 0.0147 | -0.0003 | 0.0003 | 1.3 | 1:6 |
