# Supplementary material for: New footprints from Laetoli (Tanzania) provide evidence for marked body size variation in early hominins
Source: eLife. 2016 Dec 14;5:e19568. doi: 10.7554/eLife.19568 (PMC5156529; doi:10.7554/eLife.19568)
Supplement: Supplementary file 3. — DOI: http://dx.doi.org/10.7554/eLife.19568.024 [file elife-19568-supp3.docx]

**Supplementary file 3.** Footprint imaging, measurement report 3.

Adjusted x,y,z-coordinate set of the control points.

| **ID POINT** | **X** | **Y** | **Z** |
| --- | --- | --- | --- |
| A | 0.847 | 0.000 | 1.000 |
| B | 3.412 | 0.000 | 1.050 |
| C | 3.433 | 3.274 | 0.904 |
| D | -0.004 | 3.494 | 0.779 |
|  |  |  |  |
| E | 0.000 | 0.000 | 1.000 |
| F | 2.334 | 0.000 | 1.036 |
| G | 3.332 | 2.681 | 1.054 |
| H | 0.712 | 3.885 | 1.072 |
|  |  |  |  |
| I | 0.000 | 0.000 | 1.000 |
| J | 1.335 | 0.000 | 0.979 |
| K | 1.591 | 1.562 | 1.019 |
| L | 0.170 | 1.826 | 1.051 |
|  |  |  |  |
| M | 0.122 | 0.000 | 1.000 |
| N | 2.333 | 0.000 | 1.015 |
| O | 2.303 | 3.696 | 1.097 |
| P | 0.000 | 3.619 | 1.093 |
|  |  |  |  |
