# Supplementary material for: New footprints from Laetoli (Tanzania) provide evidence for marked body size variation in early hominins
Source: eLife. 2016 Dec 14;5:e19568. doi: 10.7554/eLife.19568 (PMC5156529; doi:10.7554/eLife.19568)
Supplement: Supplementary file 4. — DOI: http://dx.doi.org/10.7554/eLife.19568.025 [file elife-19568-supp4.docx]

**Supplementary file 4.** Footprint imaging, measurement report 4.

Photoscan reports of photogrammetric processing.

| **ID DATA** | **PICTURES** (n°) | **TIE POINTS** (n° points) | **DENSE CLOUD** (n° points) | **MESH** (n° faces) | **TEXTURE** (pixel) |
| --- | --- | --- | --- | --- | --- |
| **L8** | 171 | 15,755 | 6,523,219 | 6,000,000 | 6,000 x 6,000 |
| L8/S1-1 | 31 | 4,885 | 12,788,392 | 1,000,000 | 4,096 x 4,096 |
| L8/S1-2 | 31 | 5,105 | 11,956,726 | 1,000,000 | 4,096 x 4,096 |
| L8/S1-3 | 34 | 6,721 | 14,577,445 | 1,000,000 | 4,096 x 4,096 |
| L8/S1-4 | 38 | 5,754 | 13,849,615 | 1,000,000 | 4,096 x 4,096 |
| **M9** | 277 | 16,752 | 5,520,206 | 5,000,000 | 6,000 x 6,000 |
| M9/S1-2 | 97 | 7,095 | 3,044,911 | 1,000,000 | 4,096 x 4,096 |
| M9/S1-3 | 90 | 6,695 | 3,024,744 | 1,000,000 | 4,096 x 4,096 |
| **TP2** | 180 | 14,476 | 4,803,978 | 4,000,000 | 6,000 x 6,000 |
| TP2/S2-1 | 89 | 6,326 | 9,388,424 | 1,000,000 | 4,096 x 4,096 |
| TP2/S1-1 | 55 | 4,434 | 3,624,823 | 1,000,000 | 4,096 x 4,096 |
| TP2/S1-2 | 56 | 3,991 | 4,127,016 | 1,000,000 | 4,096 x 4,096 |
| **M10** | 127 | 11,254 | 4,969,463 | 5,000,000 | 6,000 x 6,000 |
| M10/AF1 | 33 | 3,704 | 1,879,530 | 1,000,000 | 4,096 x 4,096 |
| M10/AF2 | 34 | 3,512 | 2,204,826 | 1,000,000 | 4,096 x 4,096 |
| M10/AF3 | 42 | 4,322 | 3,306,688 | 1,000,000 | 4,096 x 4,096 |
| **Site G trackway** | 117 | 3,871 | 2,968,040 | 3,000,000 | 6,000 x 6,000 |
| G2/3-18 | 30 | 6,627 | 1,584,588 | 1,000,000 | 4,096 x 4,096 |
| G1-34-35, G2/3-25-26 | 69 | 12,607 | 4,962,963 | 2,000,000 | 4,096 x 4,096 |
| G2/3-29 | 35 | 8,239 | 1,677,459 | 1,000,000 | 4,096 x 4,096 |
