# Supplementary material for: New footprints from Laetoli (Tanzania) provide evidence for marked body size variation in early hominins
Source: eLife. 2016 Dec 14;5:e19568. doi: 10.7554/eLife.19568 (PMC5156529; doi:10.7554/eLife.19568)
Supplement: Supplementary file 5. — DOI: http://dx.doi.org/10.7554/eLife.19568.026 [file elife-19568-supp5.docx]

**Supplementary file 5.** Individual fossil ages, localities and estimated statures used to build ***Figure 12***.

All ages are from ***Grabowski et al. (2015)***, unless otherwise stated. Actual femur lengths include both measurements of complete femora and length estimations based on reconstruction of incomplete bones. Actual femur lengths are from ***McHenry (1991)***, unless otherwise indicated. When the actual femur length was not available, it was estimated from the femur head diameter (FHD) (***McHenry, 1991***). Stature estimates in red were used to build ***Figure 12***. Femur measurements are in mm, statures are in cm.

| **Specimen** | **Taxon** | **Locality** | **Age** | **Actual Femur Length** | | **FHD** | **Estimated Femur Length** | **Stature McHenry (1991)** | **Stature Jungers et al. (2016)** | **Stature McHenry (1991)** | **Stature Jungers et al. (2016)** | **Notes** |
| --- | --- | --- | --- | --- | --- | --- | --- | --- | --- | --- | --- | --- |
|  |  |  |  |  |  |  |  | **Using actual femur length** | | **Using estimated femur length** | |  |
| KNM-ER 1503 | ***P. boisei*?** | Koobi Fora | 1,890 | - | | 34,5 | 343 | - | - | 128 | **129** |  |
| KNM-ER 1505 | ***P. boisei*?** | Koobi Fora | 1,890 | - | | 34,7 | 345 | - | - | 129 | **130** |  |
| KNM-ER 738 | ***P. boisei*?** | Koobi Fora | 1,880 | - | | 33,0 | 327 | - | - | 122 | **124** |  |
| KNM-ER 1500d | ***P. boisei*?** | Koobi Fora | 1,890 | 310 | | - | - | 116 | **118** | - | - | Femur length estimated by McHenry (1991) on the basis of bone reconstruction. |
| KNM-ER 993 | ***P. boisei*?** | Koobi Fora | 1,530 | 365 | | - | - | 137 | **137** | - | - | *P. boisei* in McHenry (1991). Femur length estimated by McHenry (1991) on the basis of bone reconstruction. |
| OH 80 | ***P. boisei*** | Olduvai | 1,338 | 400 | | - | - | 150 | **148** | - | - | Age and estimation of femur length are from Domínguez-Rodrigo et al. (2013). |
| SK 3155B | ***P. robustus*** | Swartkrans | 1,850 | - | | 32,4 | 320 | - | - | 120 | **122** |  |
| SK 50 | ***P. robustus*** | Swartkrans | 1,850 | - | | 41,3 | 416 | - | - | 156 | **154** |  |
| SK 82 | ***P. robustus*** | Swartkrans | 1,850 | - | | 34,5 | 343 | - | - | 128 | **129** |  |
| SK 97 | ***P. robustus*** | Swartkrans | 1,850 | - | | 36,9 | 369 | - | - | 138 | **138** |  |
| SKW 19 | ***P. robustus*** | Swartkrans | 1,850 | - | | 30,2 | 297 | - | - | 111 | **114** |  |
| SWT1/LB-2 | ***P. robustus*** | Swartkrans | 1,850 | - | | 34,4 | 342 | - | - | 128 | **129** |  |
| KSD-VP-1/1 | ***Au. afarensis*** | Woranso-Mille | 3,590 | *max* | 438 | - | - | 164 | **161** | - | - | Minimum and maximum estimations of femur length are from Haile-Selassie et al. (2010). |
|  |  |  |  | *mean* | 428 | - | - | 160 | **158** | - | - |  |
|  |  |  |  | *min* | 418 | - | - | 156 | **154** | - | - |  |
| A.L. 288-1 | ***Au. afarensis*** | Hadar | 3,200 | 280 | | - | - | 105 | **109** | - | - |  |
| A.L. 827-1 | ***Au. afarensis*** | Hadar | 3,100 | 368 | | - | - | 138 | **138** | - | - |  |
| A.L. 152-2 | ***Au. afarensis*** | Hadar | 3,350 | 324 | | 33,1 | 328 | 121 | **123** | 123 | 124 | Femur length estimated by Ward et al. (2012). |
| A.L. 333.3 | ***Au. afarensis*** | Hadar | 3,200 | 384 | | 39,5 | 397 | 144 | **143** | 148 | 147 | Femur length estimated by Ward et al. (2012). |
| Sts 14 | ***Au. africanus*** | Sterkfontein | 2,400 | - | | 29,4 | 288 | - | - | 108 | **111** |  |
| Stw 25 | ***Au. africanus*** | Sterkfontein | 2,400 | - | | 32,4 | 320 | - | - | 120 | **122** |  |
| Stw 392 | ***Au. africanus*** | Sterkfontein | 2,400 | - | | 31,5 | 311 | - | - | 116 | **119** |  |
| Stw 361 | ***Au. africanus*** | Sterkfontein | 2,400 | - | | 29,1 | 285 | - | - | 107 | **110** |  |
| Stw 403 | ***Au. africanus*** | Sterkfontein | 2,400 | - | | 31,1 | 306 | - | - | 115 | **117** |  |
| Stw 431 | ***Au. africanus*** | Sterkfontein | 2,400 | - | | 36,1 | 360 | - | - | 135 | **135** |  |
| Stw 479 | ***Au. africanus*** | Sterkfontein | 2,400 | - | | 31,0 | 305 | - | - | 114 | **117** |  |
| Stw 501 | ***Au. africanus*** | Sterkfontein | 2,400 | - | | 33,0 | 327 | - | - | 122 | **124** |  |
| Stw 31 | ***Au. africanus*** | Sterkfontein | 2,400 | - | | 30,4 | 299 | - | - | 112 | **115** |  |
| Stw 522 | ***Au. africanus*** | Sterkfontein | 2,400 | - | | 30,5 | 300 | - | - | 112 | **115** |  |
| Stw 527 | ***Au. africanus*** | Sterkfontein | 2,400 | - | | 33,0 | 327 | - | - | 122 | **124** |  |
| Stw 598 | ***Au. africanus*** | Sterkfontein | 2,200 | - | | 32,2 | 318 | - | - | 119 | **121** |  |
| MLD 17 | ***Au. africanus*** | Makapansgat | 2,715 | - | | 37,6 | 376 | - | - | 141 | **140** |  |
| MLD 25 | ***Au. africanus*** | Makapansgat | 2,715 | - | | 35,7 | 356 | - | - | 133 | **134** |  |
| MLD 46 | ***Au. africanus*** | Makapansgat | 2,715 | - | | 37,1 | 371 | - | - | 139 | **139** |  |
| BOU-VP-12/1 | ***Au. garhi*** | Bouri | 2,500 | 335 | | - | - | 125 | **127** | - | - | Femur length is from Grabowski et al. (2015). |
| MH1 | ***Au. sediba* (juv)** | Malapa | 1,977 | - | | 33,0 | 327 | - | - | 122 | **124** |  |
| MH2 | ***Au. sediba*** | Malapa | 1,977 | - | | 32,7 | 323 | - | - | 121 | **123** |  |
| OH 62 | ***H. habilis*** | Olduvai | 1,848 | 280 | | - | - | 105 | **109** | - | - | Femur length is from Grabowski et al. (2015). |
| KNM-ER 1472 | ***Homo* sp.** | Koobi Fora | 1,980 | - | | 40,2 | 404 | - | - | 151 | **150** | *H. habilis* in McHenry (1991). |
| KNM-ER 1481 | ***Homo* sp.** | Koobi Fora | 1,950 | - | | 43,0 | 435 | - | - | 163 | **160** | *H. habilis* in McHenry (1991). |
| KNM-ER 5881 | ***Homo* sp.** | Koobi Fora | 1,900 | - | | 37,0 | 370 | - | - | 138 | **138** |  |
| BSN49/P27 | ***H. erectus* s.l.** | Gona | 1,150 | - | | 32,6 | 322 | - | - | 121 | **123** |  |
| D 4167/3901 | ***H. erectus* s.l.** | Dmanisi | 1,770 | 382 | | 40,2 | 404 | 143 | **142** | 151 | 150 | Femur length is from Grabowski et al. (2015). |
| KNM-ER 736 | ***H. erectus* s.l.** | Koobi Fora | 1,580 | 482 | | - | - | 180 | **175** | - | - | *H. erectus*? in Grabowski et al. (2015). Age from Will and Stock (2015). Femur length estimated by McHenry (1991) on the basis of bone reconstruction. |
| KNM-ER 737 | ***H. erectus* s.l.?** | Koobi Fora | 1,600 | 420 | | - | - | 157 | **155** | - | - | *H. erectus* in McHenry (1991). Femur length estimated by McHenry (1991) on the basis of bone reconstruction. |
| KNM-ER 803 | ***H. erectus* s.l.** | Koobi Fora | 1,530 | 400 | | - | - | 150 | **148** | - | - | *H. erectus*? in Grabowski et al. (2015). Femur length estimated by McHenry (1991) on the basis of bone reconstruction. |
| KNM-ER 1808 | ***H. erectus* s.l.** | Koobi Fora | 1,600 | 485 | | 38,7 | 388 | 181 | **176** | 145 | 144 | Age from Will and Stock (2015). |
| KNM-WT 15000 | ***H. erectus* s.l. (juv)** | Koobi Fora | 1,470 | 432 | | 45,9 | 466 | 162 | **159** | 174 | 170 | Age from Will and Stock (2015). |
| KNM-ER 1463 | ***H. habilis/erectus or P. boisei*** | Koobi Fora | 1,530 | 310 | | - | - | 116 | **118** | - | - | *H. erectus/P. boisei* in McHenry (1991). Femur length estimated by McHenry (1991) on the basis of bone reconstruction. |
| OH 53 | ***H. habilis/erectus or P. boisei*** | Olduvai | 1,425 | 360 | | - | - | 135 | **135** | - | - | *H. habilis/P. boisei* in McHenry (1991). Femur length estimated by McHenry (1991) on the basis of bone reconstruction. |
| KNM-ER 1592 | ***H. habilis/erectus or P. boisei*** | Koobi Fora | 1,850 | 470 | | - | - | 176 | **171** | - | - | *H. habilis/P. boisei* in McHenry (1991). Femur length estimated by McHenry (1991) on the basis of bone reconstruction. |
| KNM-ER 3728 | ***H. habilis/erectus or P. boisei*** | Koobi Fora | 1,890 | 380 | | - | - | 142 | **142** | - | - | *H. habilis/P. boisei* in McHenry (1991); *P. boisei* in Wood (2011); Hominini indet. in Grabowsky et al. (2015); *H. habilis/H. rudolfensis/P. boisei* in Will and Stock (2015). Femur length estimated by McHenry (1991) on the basis of bone reconstruction. |
|  |  |  |  |  |  |  |  |  |  |  |  |  |
|  |  |  |  |  |  |  |  |  |  |  |  |  |
| **FOOTPRINTS** | | | | | | | | |  |  |  |  |
|  |  |  |  |  |  |  |  |  |  |  |  |  |
| **Specimen** | **Taxon** | **Locality** | **Age** | **Estimated stature** | | **Notes** | | |  |  |  |  |
|  |  |  |  |  |  |  |  |  |  |  |  |  |
| **-** | ***H. erectus* s.l.?** | Ileret | 1,520 | *max* | 186 | Age (range 1.53–1.51 Ma) and estimated statures are from Dingwall et al. (2013). | | |  |  |  |  |
| **-** |  |  |  | *mean* | 169 |  |  |  |  |  |  |  |
| **-** |  |  |  | *min* | 153 |  |  |  |  |  |  |  |
| **S1** | ***Au. afarensis*?** | Laetoli | 3,660 | *max* | 172 |  | | |  |  |  |  |
|  |  |  |  | *mean* | 165 |  |  |  |  |  |  |  |
|  |  |  |  | *min* | 155 |  |  |  |  |  |  |  |
| **S2** |  |  |  | 146 | |  | | |  |  |  |  |
| **G1** |  |  |  | 114 | |  | | |  |  |  |  |
| **G2** |  |  |  | 142 | |  | | |  |  |  |  |
| **G3** |  |  |  | 132 | |  | | |  |  |  |  |

**Supplementary references**

Domínguez-RodrigoM, Pickering TR, Baquedano E, Mabulla A, Mark DF, Musiba C, Bunn HT, Uribelarrea D, Smith V, Diez-Martin F, Pérez-González A, Sánchez P, Santonja M, Barboni D, Gidna A, Ashley G, Yravedra J, Heaton JL, Arriaza MC. 2013. First Partial Skeleton of a 1.34-Million-Year-Old *Paranthropus boisei* from Bed II, Olduvai Gorge, Tanzania. *PLOS ONE* **8**:e80347. doi: 10.1371/journal.pone.0080347.

Will M, Stock JT. 2015. Spatial and temporal variation of body size among early Homo. *Journal of Human Evolution* **82**:15–33. doi: 10.1016/j.jhevol.2015.02.009.

Wood B, editor. 2011. *Wiley-Blackwell Encyclopedia of Human Evolution*. Oxford and Malden: Wiley-Blackwell.
